# miR-181b-5p/SOCS2/JAK2/STAT5 axis facilitates the metastasis of hepatoblastoma

**DOI:** 10.1093/pcmedi/pbad027

**Published:** 2023-10-20

**Authors:** Yong Lv, Xiaolong Xie, Guoyou Zou, Meng Kong, Jiayin Yang, Jing Chen, Bo Xiang

**Affiliations:** Department of Pediatric Surgery and Laboratory of Pediatric Surgery, West China Hospital, Sichuan University, Chengdu 610041, China; Department of Pediatric Surgery and Laboratory of Pediatric Surgery, West China Hospital, Sichuan University, Chengdu 610041, China; Department of General Surgery, People's Hospital of Tibet Autonomous Region, Tibet 850000, China; Department of Pediatric Surgery, Children's Hospital Affiliated to Shandong University, Jinan 250022, China; Liver Transplantation Center, Department of General Surgery, West China Hospital, Sichuan University, Chengdu 610041, China; Department of Pediatric Surgery and Laboratory of Pediatric Surgery, West China Hospital, Sichuan University, Chengdu 610041, China; Department of Pediatric Surgery and Laboratory of Pediatric Surgery, West China Hospital, Sichuan University, Chengdu 610041, China

**Keywords:** miR-181b, SOCS2, metastasis, hepatoblastoma, children

## Abstract

**Introduction:**

Hepatoblastoma (HB) is a malignant liver tumor predominantly found in children and tumor metastasis is one of the main causes of poor prognosis in affected patients. The precise molecular mechanisms responsible for HB metastasis remain incompletely understood. However, there is evidence suggesting a connection between the dysregulation of microRNAs (miRNAs) and the progression of tumor metastasis in HB.

**Methods:**

The study utilized weighted gene co-expression network analysis (WGCNA) to analyze a miRNA microarray dataset of HB. The expression of miR-181b-5p in HB tissues and cells was detected using quantitative real-time PCR. The impact of miR-181b-5p on the metastatic capacity of HB was evaluated through scratch and Transwell assays. The effects of exogenously expressing miR-181b on the metastatic phenotypes of HB cells were evaluated *in vivo*. Furthermore, a luciferase reporter assay was performed to validate a potential target of miR-181b-5p in HB.

**Results:**

We found that miR-181b-5p was highly expressed in HB tissues and HB cell lines. Overexpression of miR-181b enhanced scratch healing, cell migration, and invasion abilities *in vitro*, as well as enhancing HB lung metastasis potential *in vivo*. Dual-luciferase reporter assays showed that Suppressor Of Cytokine Signaling 2 (SOCS2) was a direct target of miR-181b. The overexpression of miR-181b resulted in the suppression of SOCS2 expression, subsequently activating the epithelial–mesenchymal transition and JAK2/STAT5 signaling pathways. The rescue experiment showed that SOCS2 overexpression attenuated the effects of miR-181b on HB cells.

**Conclusion:**

Our study showed that miR-181b promotes HB metastasis by targeting SOCS2 and may be a potential therapeutic target for HB.

## Introduction

Hepatoblastoma (HB) is a rare liver tumor that primarily affects infants and young children.^1^ Pulmonary metastases are frequently observed in HB cases, with ∼20% of patients presenting with metastases in the lungs. Unfortunately, the prognosis for children with HB and pulmonary metastases is generally poor.^2^ The current standard treatment for HB pulmonary metastases involves a combination of chemotherapy and surgical resection, but <50% of patients achieve complete remission after chemotherapy.^3^ While surgical resection can effectively cure patients with primary liver tumors, those with pulmonary metastases miss out on this opportunity, significantly impacting their survival.^4^ Moreover, chemotherapy regimens for HB patients with pulmonary metastases may involve the administration of multiple agents such as cisplatin, etoposide, and doxorubicin. These treatments necessitate long-term chemotherapy and can result in serious toxic side effects.^5^ Metastasis of HB is a major obstacle to effective clinical treatment due to the incomplete understanding of its key biological pathways and related functional genes. Further exploration and research by pediatric surgeons are needed to address this issue.

Weighted gene co-expression network analysis (WGCNA) is a bioinformatics tool used to study the structure and function of large-scale biomolecular networks. The interactions between biomolecules form large-scale and complex biomolecular networks, and the analysis of the structure and function of these networks is crucial for understanding complex biological problems. Identifying key nodes in the network system has always been an important issue in systems biology.^6^ Since microRNAs (miRNAs) are involved in the development of many diseases, the discovery of disease-related molecules or regulatory mechanisms can be facilitated by miRNA–mRNA networks.^7^ WGCNA can be used to identify signature genes or hub genes within modules to reveal gene interactions and regulatory mechanisms.^8^ A previous study used WGCNA to analyze RNA sequencing data and miRNAs expression profile data to identify a number of biomarkers for early tumor diagnosis.^9^ We collected miRNA expression profile data and predicted HB metastasis-related miRNA, and performed experimental validation and mechanistic analysis.

We identified miR-181b-5p, which exhibits a high correlation with HB metastasis, through the application of WGCNA. miR-181b-5p is a multifunctional miRNA that has been reported to participate in regulating the malignant biological behavior of various pediatric solid tumors. Abnormal expression of miR-181 family members was detected in 32 children with neuroblastoma, and overexpression of miR-181b *in vitro* significantly induced the growth and invasion of neuroblastoma cells.^10^ Ewing's sarcoma is a common primary malignant bone tumor in children. A miRNA expression profile analysis found that miR-181b is abnormally expressed in the tumor and may serve as a potential prognostic marker for this invasive pediatric bone tumor.^11^ These studies suggest that miR-181b plays an important regulatory role in the development of pediatric tumors. Our bioinformatics analysis revealed that miR-181b may play a potential role in HB metastasis. However, the exact mechanism through which miR-181b influences the progression of HB remains unclear, and the specific role of miR-181b in HB metastasis is also not well understood. This study aims to investigate the effect and molecular mechanism of miR-181b in HB metastasis, in order to provide a theoretical basis for the development of new strategies for the prevention and treatment of HB metastasis.

## Methods

### WGCNA

WGCNA was conducted using the miRNA expression profile data from the GSE153089 dataset. The tissue of this dataset included nontumorous liver tissue surrounding HB (*n* = 14), primary HB tumor tissues of fetal subtype (*n* = 11) and embryonal subtype (*n* = 10), and metastatic HB tumor tissue (*n* = 9). We used the WGCNA function package in R software to screen for gene modules associated with disease phenotypes and further analyzed the relationship between these modules and HB tumor samples. The WGCNA function was utilized to remove unqualified genes and samples, resulting in the construction of a scale-free co-expression network. The adjacency between genes was calculated using the “soft” threshold power (β), which was then transformed into a topological overlap matrix (TOM). This matrix helped measure network connectivity and similarity. Based on the dissimilarity of TOM and a minimum gene number map (*n* = 30), genes with similar expression profiles were grouped into gene modules using average linkage hierarchical clustering and dynamic tree-cutting function detection. To further analyze the modules, the dissimilarity of module eigen genes was calculated. A cut line for the module dendrogram was chosen, leading to the merging of some modules.^12^ We quantify associations of individual genes with our trait of interest (tumor metastasis) by defining gene significance (GS) as the correlation between the gene and the trait. The module membership (MM) was calculated as the correlation between the gene expression profile and the module eigengene. This measures the degree of membership of each gene within a module. This allows us to quantify the similarity of all genes on the array to every module. Differentially expressed genes (DEGs) between HB and normal liver were screened from GSE153089 with the “edgeR” package. Significantly changed genes were selected with *P* value < 0.05 and log_2_|fold-change|≥1.

### Tissue materials

We consecutively collected the data and tissues of 12 patients with HB who underwent hepatectomy at our institution between January 2021 and January 2023. The Ethics Committee on Biomedical Research, West China Hospital of Sichuan University approved this study (2019–1085). With consent from patients or parents/legal guardians, tumor tissue and adjacent normal liver tissues were collected from patients with a confirmed diagnosis of HB. Collected tissues were stored in liquid nitrogen prior to molecular analysis. Informed, signed consent with regard to the collection and use of sample tissues in this study was obtained in advance from parents/legal guardians of the patients. The collection of samples was approved by the Ethics Committee of the West China Hospital of Sichuan University. All information regarding the human material was managed using anonymous numerical codes and the experiments were conducted following the Helsinki declaration.

### Cell culture and transfection

The HB cell lines Huh6 and HepG2, and normal hepatocytes L02 were cultured according to ATCC recommended culture methods. HB cells were inoculated into 6-well culture plates at 1 × 10^4^ cells/well, and when the cell fusion reached 70%–80%, the cells were transfected according to the instructions of the Lipofectamine 8000 transfection kit. To detect the effect of miR-181b-5p and Suppressor Of Cytokine Signaling 2 (SOCS2) on HB cells, HB cells were transfected with miR-181b-5p mimic, miR-181b-5p inhibitor, pCS2-SOCS2, SOCS2 Small interfering RNA (siRNAs), and negative controls, respectively. The miR-181b mimic (Product ID: miR10000257-1-5) and miR-181b inhibitor (Product ID: miR20000257-1-5) were purchased from RIBOBIO (Guangzhou, China). The siRNA was purchased from Tsingke Biotech (Beijing, China).

### Quantitative real-time PCR

The total RNA of each group of cells was extracted separately according to the instructions of the Total RNA Extraction Kit (TRIzol), and the quality of total RNA of each group was evaluated using a Nanodrop spectrophotometer. Reverse transcription was performed using 2 μg of RNA as template. After the cDNA was obtained by reverse transcription, the real-time PCR reactions were performed in the SYBR Green One-Step Quantitative real-time PCR (qRT-PCR) Kit. The results were analyzed by the 2^−△△CT^ method.

### Western blot

To extract total protein from transfected cells in each group, lysis buffer was used. The protein samples were then incubated at 95°C for 15 min to denature the proteins. The denatured proteins were loaded onto a 10% SDS polyacrylamide gel for electrophoresis and separation. The separated proteins were transferred onto a membrane, which was then blocked with a 5% skimmed milk solution for 1 h at room temperature. The membrane was subsequently incubated with the primary antibody overnight. After washing the membrane, it was incubated with the secondary antibody for 1 h. Finally, the membrane was exposed using an Electrogenerated chemiluminescence system.

### Scratch assay

Huh6 and HepG2 cells were diluted with serum-free dulbecco's modified eagle medium to a concentration of 2 × 10^4^/ml and inoculated in 6-well plates. When the cell confluence reached 100%, a 200 μl pipette was used to make a scratch. After 48 h of continuous culture, the migration distance of cells was observed and recorded under a microscope.

### Transwell assay

The transfected HB cells were counted and 10^5^ cells were added to the upper Transwell chamber. Next, 500 μl of fetal bovine serum was added to the lower chamber. Then the chamber was incubated for 48 h before fixing and staining the cells with Giemsa stain. The number of cells that had passed through the membrane was counted by microscope.

### Double-luciferase reporter assay

The miR-181b control plasmid and miR-181b overexpression plasmid were co-transfected with the 3'-UTR (untranslated region) wild-type (WT-SOCS2-3'-UTR) and mutant (MUT-SOCS2-3'-UTR) SOCS2 vector into Huh6 cells. After 48 h of transfection, the supernatant was aspirated and discarded, and the cells were washed twice with PBS, followed by lysis with cell lysis buffer for 15 min, and the cell lysate was collected. Specifically, 100 μl of luciferase reaction reagent was added to an opaque 96-well plate, followed by the addition of 20 μl of cell lysate. The plate was then shaken and the activity of the luciferase reporter gene was measured using a luminometer. Next, 100 μl of another reagent, Renilla luciferase reaction reagent, was added to the same plate, and shaken, and the activity was measured.

### Formation of lung metastases *in vivo*

The animal ethics committee of West China Hospital of Sichuan University approved the animal studies (Approval Number: 20 230 106 004). To explore the role of miR-181b *in vivo*, miR-181b-overexpressed and Green fluorescent protein(GFP)-labeled lentivirus (LV-miR-181b) and control lentivirus (LV-NC) were transfected into Huh6 cells. A total of 10 nude mice were randomly divided into LV-NC group (*n* = 5) and LV-miR-181b group (*n* = 5). Then the Huh6 cells (2 × 10^5^/200 μl) were injected into the nude mice via tail vein. After 4 weeks, the mice were euthanized and the lungs were removed for imaging. An IVIS^®^ Lumina III *in vivo* imaging system was used to monitor for lung metastasis, imaged *ex vivo*. GFP luminescence was quantified as average radiance using Living Image software version 4.0 and the region of interest (ROI) tool.

### Statistical analysis

SPSS23.0 statistical software was applied for statistical analysis, t-test was applied to compare two groups, and one-way one-way analysis of variance (ANOVA) was used for comparison between multiple groups; *P* < 0.05 indicates that the results of comparison between experimental groups are statistically significant. All data are shown as mean ± SD from more than three independent experiments. Data were analyzed by Graph Pad Prism Version 9.0 software.

## Results

### WGCNA screening for miRNAs associated with HB metastasis

**Figure 1.**
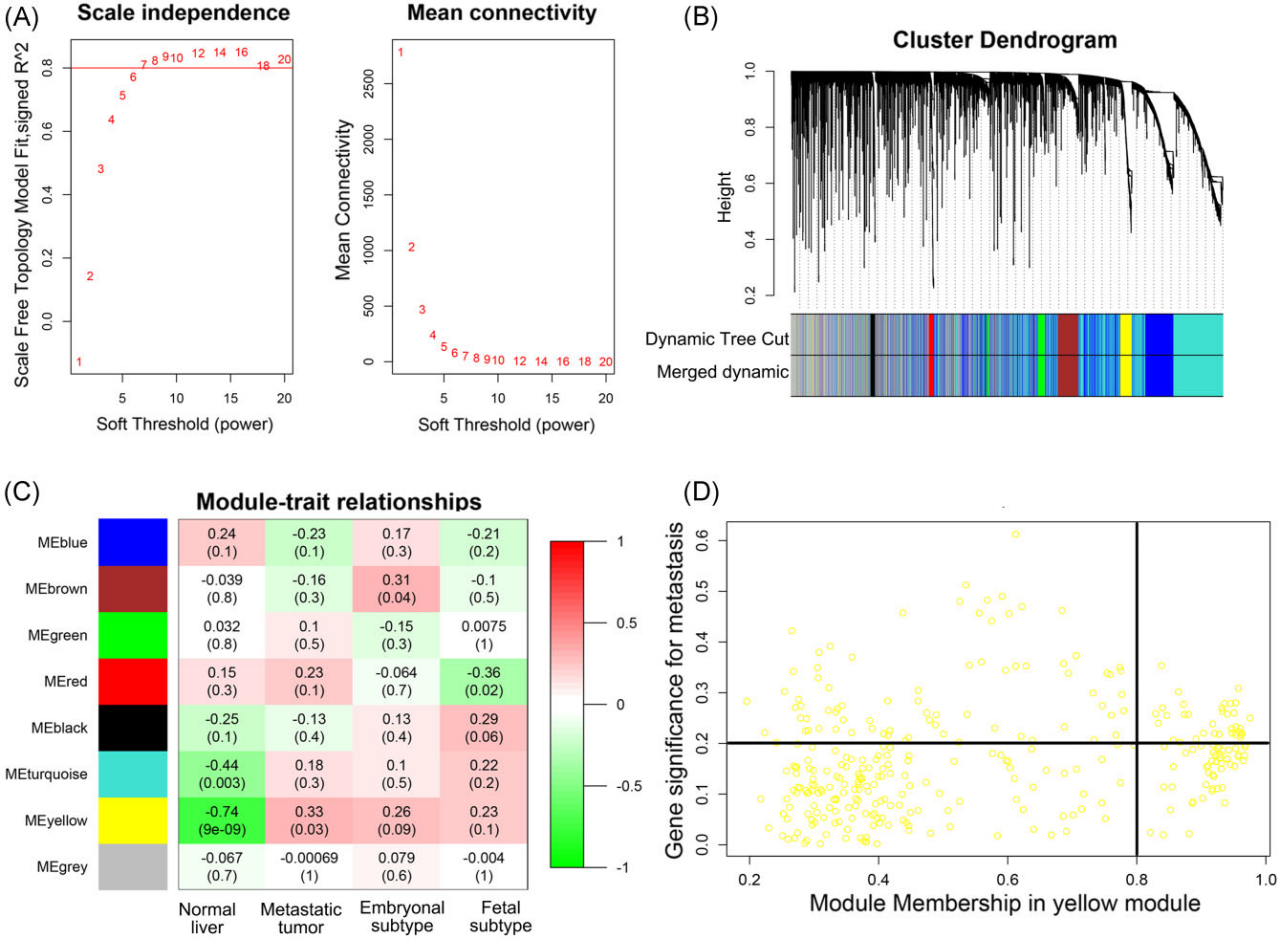
Weighted gene co-expression network analysis to identify HB metastasis-related miRNAs. (**A**) Analysis of network topology for various soft-thresholding powers. The left panel shows the scale-free fit index (*y*-axis) as a function of the soft-thresholding power (*x*-axis). The right panel displays the mean connectivity (degree, *y*-axis) as a function of the soft-thresholding power (*x*-axis). (**B**) Cluster dendrograms representing groups of genes identified using weighted gene co-expression network analysis in HB datasets with the assigned module colors. (**C**) Module–trait relationship of the significant modules correlating to HB, with correlation values to metastasis, embryonal, and fetal subtype phenotype. Each row corresponds to a module eigengene and each column corresponds to a trait. Each cell contains the corresponding correlation and *P*-value. The figure is color-coded by correlation according to the color legend. (**D**) A scatterplot of GS for metastasis versus MM in the yellow module.

We conducted WGCNA on the microRNA expression profile. When β = 7 was set as the soft threshold, the weighted gene co-expression network conformed to a scale-free network (Fig. 1A). We constructed a systematic clustering tree and topological overlap matrix for miRNAs, identifying a series of modules formed by highly interconnected miRNAs (Fig. 1B). We found that the yellow module (*r* = 0.33, *P* = 0.03) was closely related to HB metastasis (Fig. 1C), so we extracted the miRNAs from the yellow molecular module for subsequent analysis. We selected miRNAs with MM > 0.8 and GS > 0.2 in the yellow module as candidate key miRNAs (Fig. 1D). To further refine our investigation, we intersected the differentially expressed miRNAs in GSE153089 with the candidate miRNAs obtained from WGCNA, resulting in a selection of 22 miRNAs. Notably, miR-181b-5p exhibited the highest MM value of 0.97). In the context of identifying core miRNA, MM can be used to prioritize miRNAs that have a strong correlation with a specific module of interest, so we focused on miR-181b-5p (Table 1).

**Table 1.** HB metastasis-related miRNAs.

miR name	GS	MM yellow	logFC	Adjusted *P* value
hsa-miR-181b-5p	0.250 020 918	0.975 317 735	2.9 712 381	5.91E-04
hsa-miR-369–5p	0.270 138 284	0.839 927 235	1.398 714 286	8.22E-03
hsa-miR-370–3p	0.20 216 469	0.866 726 772	3.427 380 952	4.62E-07
hsa-miR-379–5p	0.220 281 129	0.903 251 165	4.549 190 476	2.80E-06
hsa-miR-381–5p	0.258 657 861	0.829 147 093	1.312 952 381	1.36E-03
hsa-miR-382–5p	0.208 796 816	0.891 768 152	4.399 857 143	2.10E-07
hsa-miR-323a-3p	0.27 849 034	0.935 748 109	3.010 761 905	3.52E-04
hsa-miR-431–3p	0.260 110 953	0.950 741 252	3.522 904 762	9.31E-07
hsa-miR-433–3p	0.26 322 071	0.932 935 631	4.626 095 238	3.62E-07
hsa-miR-329–3p	0.222 315 628	0.958 981 262	2.82 147 619	9.24E-05
hsa-miR-410–3p	0.278 808 843	0.945 555 087	2.935 428 571	3.93E-05
hsa-miR-493–5p	0.229 789 353	0.960 337 525	3.78	9.57E-06
hsa-miR-493–3p	0.219 797 894	0.96 321 338	5.319 666 667	4.95E-08
hsa-miR-432–5p	0.217 818 375	0.927 788 409	5.647 809 524	4.22E-07
hsa-miR-495–3p	0.232 811 267	0.953 490 196	3.768 285 714	3.69E-07
hsa-miR-539–5p	0.256 961 968	0.873 771 468	1.843 904 762	3.09E-03
hsa-miR-411–5p	0.217 439 679	0.952 964 818	3.847 952 381	4.79E-06
hsa-miR-411–3p	0.269 745 634	0.932 166 157	2.671 904 762	6.07E-04
hsa-miR-758–5p	0.239 749 502	0.833 314 769	1.495 380 952	2.12E-02
hsa-miR-758–3p	0.308 512 376	0.957 311 474	3.454 619 048	2.22E-06
hsa-miR-668–3p	0.276 947 318	0.872 343 941	1.829 285 714	5.47E-03
hsa-miR-543	0.229 067 693	0.946 750 121	3.768 285 714	1.52E-06

### miR-181b promotes the migration and invasion of HB cells

**Figure 2.**
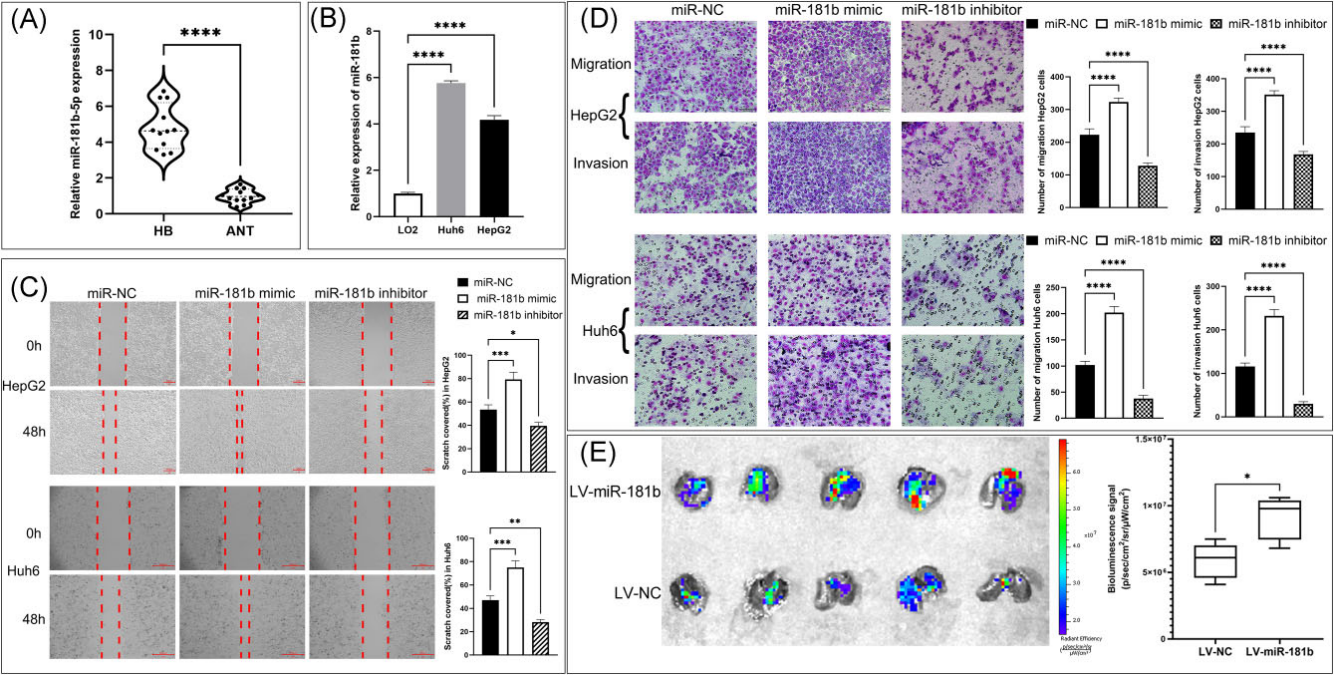
miR-181b-5p was upregulated in HB and promoted migration and invasion. (**A**) miR-181b-5p expression in human hepatoblastoma (HB, *n* = 12) tissues and adjacent normal tissues (ANT, *n* = 12) was determined by qRT-PCR. The relative expression levels of miR-181b-5p in HB were higher than those in ANT. The violin plot displays the distribution of a continuous variable across different categories. ^****^P < 0.0001 by Student's t-test. (**B**) Expression levels of miR-181b-5p in HB cells (Huh6 and HepG2) and LO2 cells. Three repetitions were carried out and data are presented as means ± SD. ^****^P < 0.0001 by one-way analysis of variance. (**C**) Wound-healing assay analysis of HepG2 and Huh6 cells treated with either miR-181b-5p mimics or miR-181b-5p inhibitor versus scramble controls for 48 h (left: representative brightfield pictures of cells at 0 or 48 h time-point; right: quantification of cells migrated into wound area). The width of the gap between two patches of cells was measured, scratch covered rate was calculated, and any significant differences between the control group and treatment group were identified. All quantifications were done with three independent repeats. Data are presented as means ± SD. *P < 0.05, **P < 0.01, ***P < 0.001 by one-way analysis of variance. (**D**) Transwell assay analysis of HepG2 and Huh6 cells treated with either miR-181b-5p mimic or miR-181b-5p inhibitor versus scramble controls for 48 h (left: representative pictures of Giemsa-stained migrated and invaded cells at 48 h time-point; right: bar graphs show the number of migrated and invaded cells through the membrane). The number of migrated and invaded cells was calculated and any significant differences between the control group and treatment group were identified. All quantifications were done with three independent repeats. Data are presented as means ± SD. ^****^P < 0.0001 by one-way analysis of variance. (**E**) Representative fluorescence pictures of *ex vivo* lungs shown at completion of the 4-week study period (left: GFP fluorescent images of lung metastases at the endpoint; right: the box plot shows the average bioluminescence signal measured in the lungs). The average radiant efficiency measured in the lungs of LV-miR-181b group mice was higher than in lungs of LV-NC group mice (*n* = 5 per group). Data are presented as means ± SD. *P < 0.05 by Student's t-test.

We examined the expression of miR-181b in 12 HB tissues and adjacent normal liver tissues. The qRT-PCR showed that miR-181b was significantly upregulated in HB tissue (Fig. 2A). Using human liver cell L02 as a control group, qRT-PCR revealed that miR-181b expression was higher in HB cells than in control cells (Fig. 2B). To further investigate the function of miR-181b in HB cell migration and invasion, scratch and Transwell assays were performed. The scratch assay showed that upregulation of miR-181b significantly accelerated wound closure in HepG2 and HuH6 cells, while miR-181b knockdown decreased the wound closure capacity (Fig. 2C). Consistently, Transwell assays demonstrated that overexpression of miR-181b significantly promoted the migration and invasion of HB cells, whereas downregulation of miR-181b significantly inhibited the migratory and invasive ability of HB cells (Fig. 2D). Since miR-181b enhanced migration and invasion of HB cells, we aimed to explore whether miR-181b could promote HB lung metastasis *in vivo*. We generated stable clones of GFP-labeled Huh6 cells either with miR-181b overexpressed lentivirus (LV-miR-181b) or control lentivirus only (LV-NC). Then these cells were injected into nude mice via tail vein and fluorescence signals in the *ex vivo* lungs were measured after 4 weeks. We found that lungs from the LV-miR-181b group had a higher fluorescence signal than those from the LV-NC group. These data suggest that miR-181b enhanced Huh6 cell metastasis *in vivo* (Fig. 2E).

### miR-181b promotes epithelial–mesenchymal transition in HB cells

**Figure 3.**
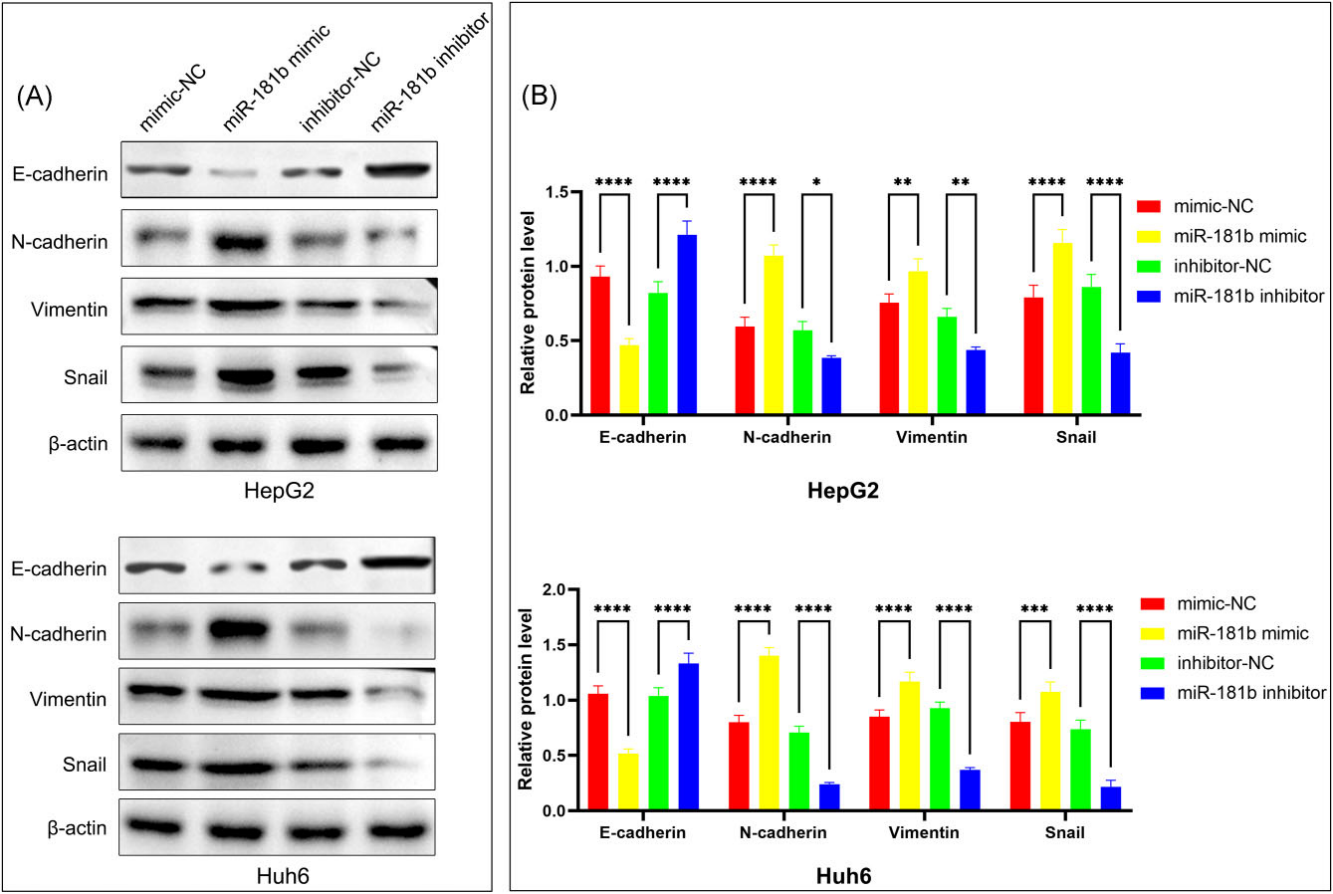
miR-181b-5p promotes HB EMT. (**A**) Western blot analysis of E-cadherin, N-cadherin, vimentin, and snail in HB cells after transfection with miR-181b-5p mimic, inhibitor, or negative control. (**B**) Quantification of the Western blot: protein expression was quantified by band intensity and normalized to β-actin, values are expressed as mean ± SD (*n* = 3). *P < 0.05, **P < 0.01, ***P < 0.001, ^****^P < 0.0001 by one-way analysis of variance.

Tumor cells undergoing epithelial–mesenchymal transition (EMT) are more prone to invasion and metastasis. We investigated the relationship between miR-181b and EMT in HB cells. Western blot demonstrated that overexpression of miR-181b decreased the expression of epithelial marker E-cadherin but increased the expression level of mesenchymal markers N-cadherin, vimentin, and snail in the HB cells. Conversely, miR-181b inhibitor yielded the opposite effect on these EMT marker (Fig. 3). These *in vitro* experiments suggest that miR-181b promotes EMT in HB cells.

### SOCS2 is a direct target of miR-181b-5p

**Figure 4.**
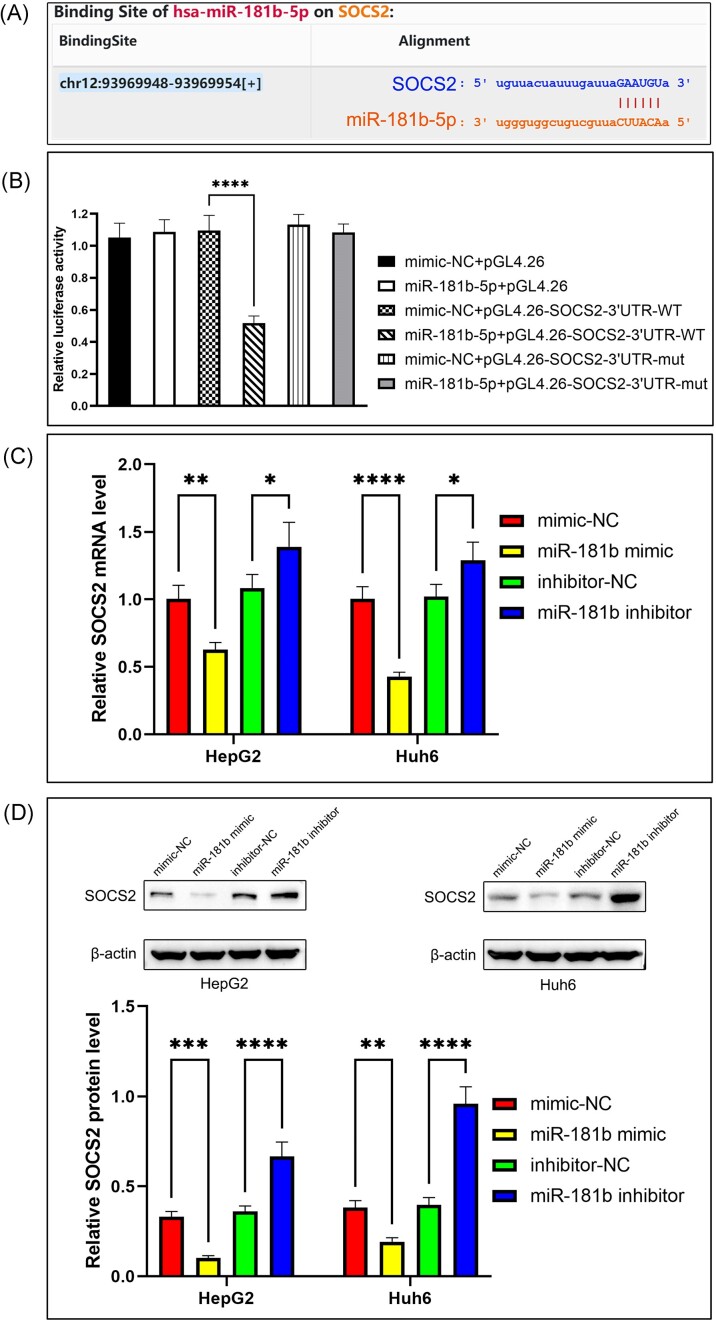
SOCS2 is a direct target of miR-181b-5p. (**A**) The seed sequence of miR-181b-5p is complementary to the targeting site in the 3′UTR of SOCS2. (**B**) Dual-luciferase assay validating SOCS2 as a target of miR-181b-5p. Luciferase reporter assay was performed in Huh6 cells using the synthetic miR-Control or miR-181–5p in combination with the WT or the MUT 3′UTR of the SOCS2 construct. All quantifications were done with three independent repeats. Data are represented as means ± SD. ^****^P < 0.001 by one-way analysis of variance. (**C**) Expression of SOCS2 mRNA in HB cells was assessed following alteration of miR-181b-5p expression using qRT-PCR. All quantifications were done with three independent repeats, and the expression of Glyceraldehyde -3-phosphate dehydrogenase (GAPDH) was used as a PCR internal reference. Data are represented as means ± SD. *P < 0.05, **P < 0.01, ^****^P < 0.0001 by one-way analysis of variance. (**D**) Expression of SOCS2 protein in HB cells was analyzed by western blot following alteration of miR-181b-5p expression. Protein expression was quantified by band intensity and normalized to β-actin, values are expressed as mean ± SD (*n* = 3). **P < 0.01, ***P < 0.001, ^****^P < 0.0001 by one-way analysis of variance.

To explore the underlying mechanism of miR-181b-5p, we conducted bioinformatics, and the results from the web server Starbase showed that miR-181b complementarily binds to the 3'-UTR of SOCS2 (Fig. 4A). SOCS2 is downregulated in various human tumors and is associated with tumor metastasis and progression.^13^ However, there are no reports on the expression of SOCS2 in HB, nor are there any reports on its targeting relationship with miR-181b. To verify whether SOCS2 was a direct target of miR-181b-5p, we performed a dual-luciferase reporter assay on Huh6 cells. The results showed that miR-181b mimic specifically inhibited the luciferase activity of the wild-type (WT) SOCS2-3'UTR reporter plasmid in Huh6 cells, while it had no significant effect on the luciferase activity of the mutant (MUT) SOCS2-3'UTR reporter (Fig. 4B). Next, we used Western blot and qRT-PCR to investigate the effect of miR-181b on endogenous SOCS2 expression. After transfection with miR-181b-mimic in HB cells, the expression of SOCS2 reduced significantly, at both mRNA and protein level. Conversely, miR-181b-inhibitor transfection led to the upregulation of SOCS2 expression (Fig. 4C,D). These results indicate that miR-181b has the potential to directly target and suppress the expression of SOCS2 in HB.

### Inhibition of SOCS2 expression accounts for miR-181b-5p function in HB

**Figure 5.**
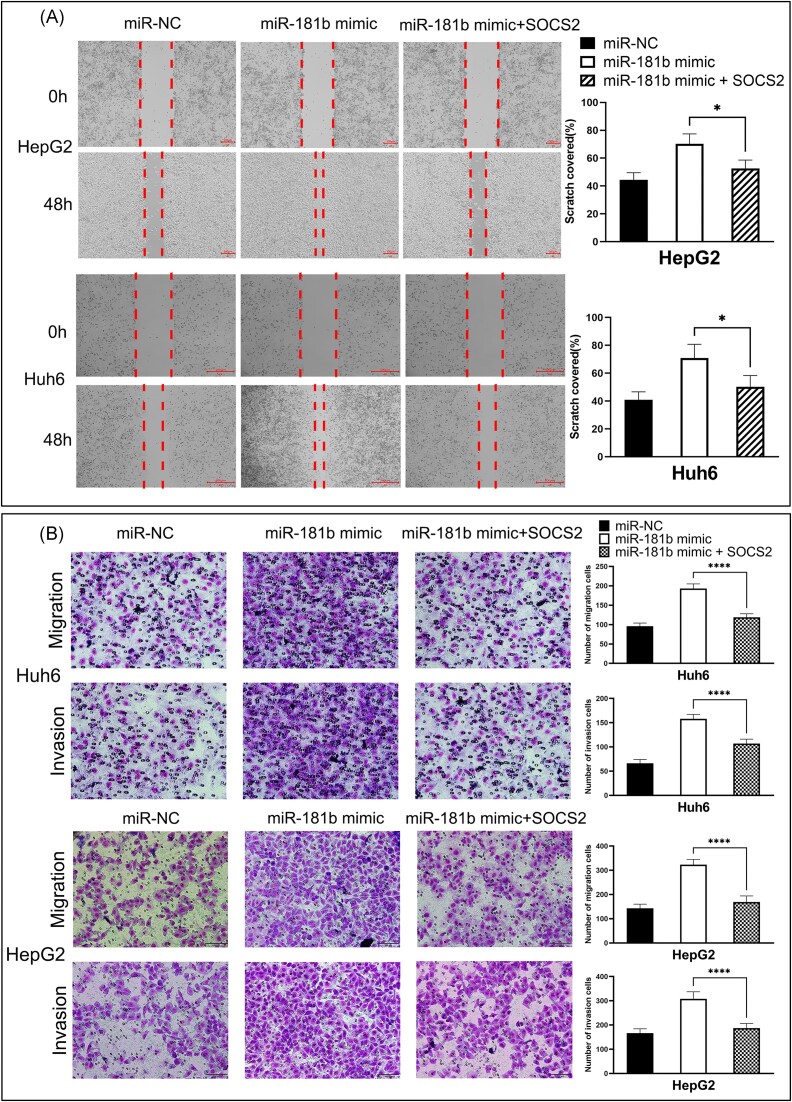
The effects of miR-181b-5p on HB cell migration and invasion were partially reversed by overexpression of SOCS2. (**A**) Wound healing assays were conducted to evaluate the motility of HB cells transfected with miR-181b-5p mimic alone or co-transfected with SOCS2. (Left: representative brightfield pictures of cells at 0 or 48 h time-point; right: quantification of cells migrated into wound area). The width of the gap between two patches of cells was measured and scratch cover rate was calculated. All quantifications were done with three independent repeats. Data are presented as means ± SD. *P < 0.05 by one-way analysis of variance. (**B**) Transwell assays were conducted in HB cells transfected with miR-181b-5p mimic alone or co-transfected with SOCS2. (Left: representative pictures of Giemsa-stained migrated and invaded cells at 48 h time-point; right: bar graphs show the number of migrated and invaded cells through the membrane). The number of migrated and invaded cells was calculated. All quantifications were done with three independent repeats. Data are represented as means ± SD. ^****^P < 0.0001 by one-way analysis of variance.

To further verify whether the effects of miR-181b-5p on HB were mediated by SOCS2, we conducted rescue experiments. Overexpression of miR-181b significantly enhanced HB cell migration, while co-transfection with SOCS2 overexpression plasmid (only the Coding sequence of SOCS2 was expressed, without the miRNA-181b targeting site in the 3′UTR) reversed this effect (Fig. 5A). Moreover, transwell assay indicated that additional SOCS2 abrogated the promotion of migration and invasion of HB cells induced by miR-181b mimic (Fig. 5B). These results indicate that miR-181b-5p might promote HB cell migration and invasion through SOCS2.

### miR-181b-5p enhanced the activation of EMT via the JAK2/STAT5 pathway

**Figure 6.**
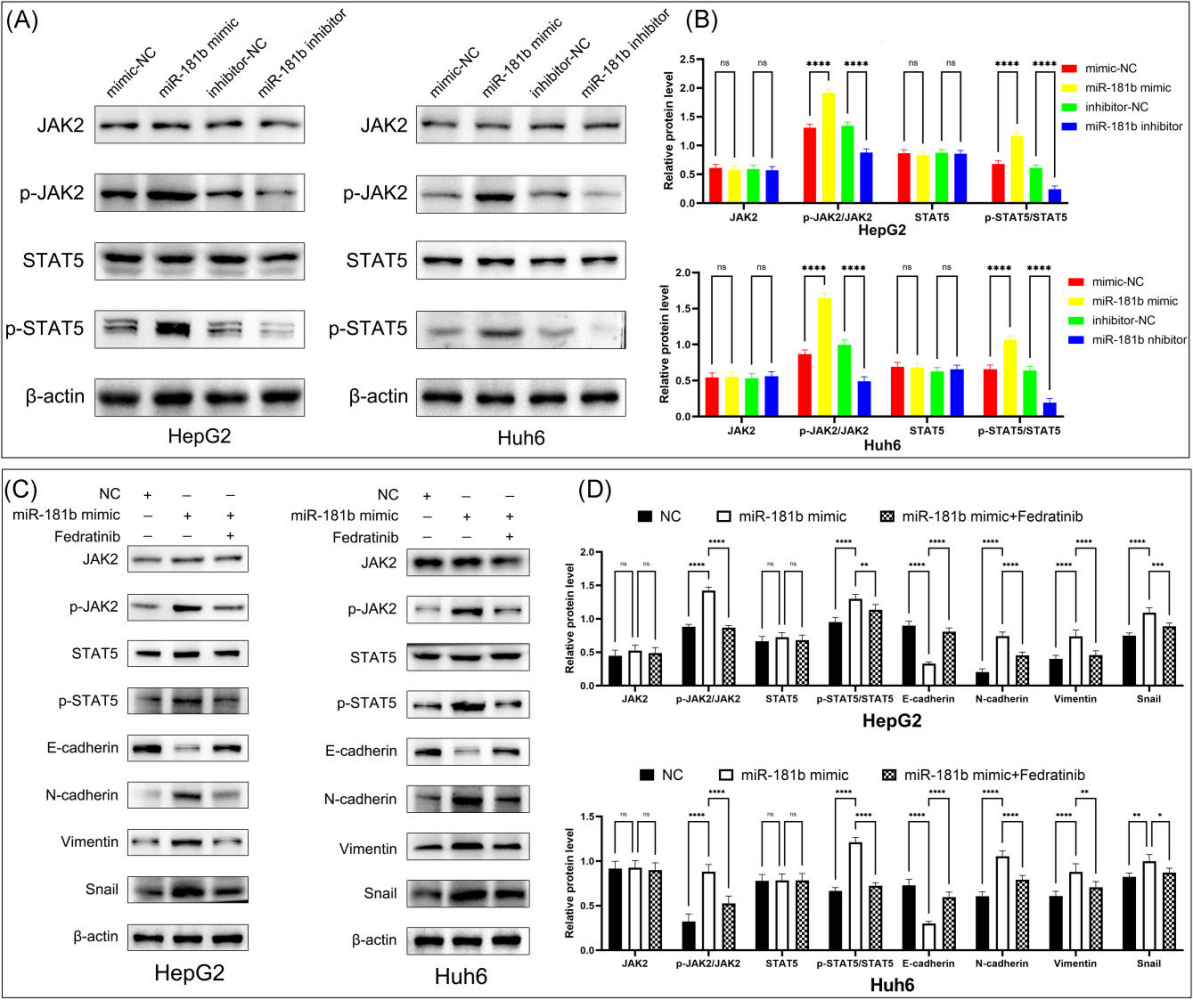
miR-181b-5p enhanced the activation of the JAK2/STAT5 pathway and EMT in HB. (**A**) Western blot of JAK2, p-JAK2, STAT5, and p-STAT5 on HB cells transfected with miR-181b-5p mimic, inhibitor, or negative control. (**B**) Quantitation of JAK2, p-JAK2, STAT5, and p-STAT5 protein using ImageJ software. Protein expression was quantified by band intensity and normalized to β-actin, values are expressed as mean ± SD (*n* = 3). ^****^P < 0.0001 by one-way analysis of variance. (**C**) Western blot of JAK2, p-JAK2, STAT5, p-STAT5, E-cadherin, N-cadherin, vimentin, and snail protein on HB cells overexpressing miR-181b or control vector and treated with Fedratinib. (**D**) Quantitation of JAK2, p-JAK2, STAT5, p-STAT5, E-cadherin, N-cadherin, vimentin, and snail protein using ImageJ software. Protein expression was quantified by band intensity and normalized to β-actin, values are expressed as mean ± SD (*n* = 3). ^****^P < 0.0001 by one-way analysis of variance. ns, Not significant.

It has been reported that SOCS2 is a negative regulator of JAK2/STAT5 signaling pathways, which play a critical role in cell EMT.^14,15^ To better understand the relationship between miR-181b, SOCS2, and downstream signaling pathways, we conducted a series of assays. Western blot results showed that overexpression of miR-181b enhanced the phosphorylation levels of JAK2 and STAT5, whereas miR-181b inhibitor decreased the expression of p-JAK2 and p-STAT5 in HB cells (Fig. 6A). These results suggest that miR-181b activates the JAK2/STAT5 pathway. We further explored the functional significance of JAK2/STAT5 signaling in the pro-metastasis role of miR-181b-5p in HB cells using the JAK2/STAT5 signaling inhibitor Fedratinib (TG101348). Western blot results revealed that the stimulatory effect of miR-181b-5p on EMT and JAK2/STAT5 activity was attenuated by Fedratinib (Fig. 6C), indicating that Fedratinib could reverse the effect of miR-181b on EMT in HB cells, via targeting the JAK2/STAT5 signaling pathway.

### The miR-181b-5p/SOCS2/JAK2/STAT5 axis regulated HB metastasis

**Figure 7.**
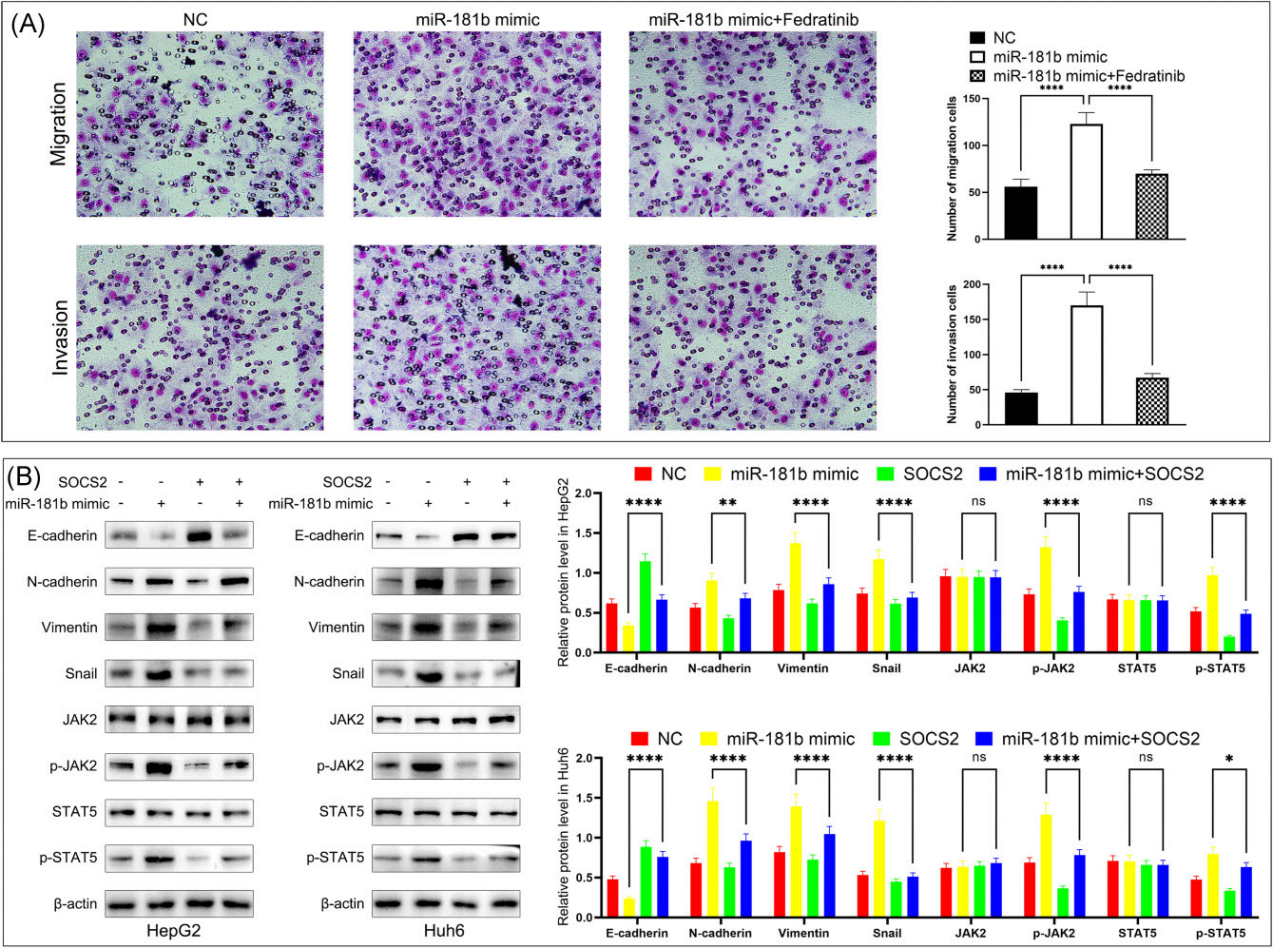
The miR-181b-5p/SOCS2/JAK2/STAT5 axis regulated HB migration and invasion. (**A**) Fedratinib decreased the migration and invasion of Huh6 cells in the miR-181b-5p overexpression group (left: representative pictures of Giemsa-stained migrated and invaded cells at 48 h time-point; right: the bar graphs show the number of migrated and invaded cells through the membrane). All quantifications were performed with three independent repeats, data are presented as means ± SD. ^****^P < 0.0001 by one-way analysis of variance. (**B**) Western blot analysis (left) and quantification (right) were performed to detect protein levels of JAK2, p-JAK2, STAT5, p-STAT5, E-cadherin, N-cadherin, vimentin, and snail in HB cells with indicated modifications. All quantifications were performed with three independent repeats, data are presented as means ± SD. *P < 0.05, **P < 0.01, ^****^P < 0.0001 by one-way analysis of variance. ns, Not significant.

To investigate whether the pro-metastasis role of miR-181b-5p in HB cells was caused by the activation of the JAK2/STAT5 pathway, we employed a JAK2 inhibitor, Fedratinib. Transwell assays showed that Fedratinib inhibited miR-181b-induced increases in HB cell migration and invasion (Fig. 7A). In addition, overexpression of SOCS2 partially reversed the promotion of JAK2/STAT5 and EMT activity by miR-181b-mimic in HB cells (Fig. 7B). The above results suggest that miR-181b-5p directly targets SOCS2, resulting in activation of JAK2/STAT5 signaling in HB cells.

## Discussion

The incidence of metastasis is a critical factor affecting the prognosis and survival of children with HB. Therefore, a comprehensive understanding of the underlying mechanisms of HB metastasis and identifying novel therapeutic targets is vital for improving the clinical outcomes of HB children. Our study showed that dysregulation of the miR-181b/SOCS2/JAK2/STAT5 axis is associated with metastasis of HB. Specifically, we found that upregulation of miR-181b in HB cells promotes tumor metastasis by inhibiting the expression of SOCS2, which leads to activation of JAK2/STAT5 signaling and subsequent promotion of EMT related to invasion. Conversely, restoration of SOCS2 expression or inhibition of JAK2/STAT5 signaling can suppress tumor metastasis in HB. Taken together, our results suggest that the miR-181b/SOCS2/JAK2/STAT5 axis may represent a novel therapeutic target for the prevention and treatment of HB.

Identifying and characterizing the function of the miR-181b/SOCS2 axis in HB metastasis is significant. Firstly, as HB is typically asymptomatic in the early stages, understanding the role of the miR-181b/SOCS2 axis in this process may provide new insights into the mechanisms underlying HB progression and metastasis. Targeting this signal axis may be an effective strategy to treat metastatic disease in children with HB. Secondly, the miR-181b/SOCS2 axis is not known to regulate HB cell metastasis in previous studies. Therefore, our data may shed light on the molecular mechanisms that drive HB metastasis and identify potential novel therapeutic targets. Thirdly, identifying the function of miR-181b in HB may have broader implications for cancer research and therapy. Dysregulation of miRNA expression is a hallmark of many cancers, and miRNAs may serve as potential diagnostic or prognostic biomarkers as well as therapeutic targets. The miR-181 family is a highly conserved group of miRNAs that are increasingly important in biomedical research, and these miRNAs exhibit aberrant expression patterns in different solid tumors, where they can function as either tumor suppressors or promoters of cancer.^16^ One study found that the TGFβ pathway regulates the transcription of miR-181b, which in turn promotes the progress and chemoresistance of liver cancer.^17^ Neuroblastoma is a solid tumor that predominantly affects children. There is an upregulation of miR-181b in neuroblastoma, and this dysregulation is significantly associated with the prognosis of children with neuroblastoma.^18^ Another important finding is that miR-181b was up-regulated in MYCN-amplified neuroblastoma relative to the other tumor subtypes, while being decreased in retinoic acid-induced neuroblastoma.^19^ In contrast to earlier findings, however, some studies have shown that miR-181b has anti-cancer effects in other tumors. In glioma, miR-181b acts as a tumor suppressor by inhibiting tumor growth and invasion and inducing apoptosis, possibly through targeting the NF-κB and EMT pathways.^20^ Characterizing the role of miRNAs like miR-181b in HB may lead to the development of more effective and personalized therapies for a wide range of malignancies.

In our study, miR-181b has been shown to target SOCS2, a negative regulator of the JAK2/STAT5 signaling pathway. The JAK2/STAT5 signaling pathway is a crucial intracellular signaling pathway that plays a significant role in a variety of biological processes, including cell proliferation, differentiation, and survival.^21^ Growing evidence suggests that dysregulation of the JAK/STAT pathway is associated with tumor cell proliferation, migration, and invasion.^22^ In the liver, the SOCS2/JAK2/STAT5 axis and the negative regulatory role of SOCS2 are crucial for maintaining the normal physiological status of liver cells.^23^ Previous studies have shown that high-glucose-induced JAK/STAT activation upregulates the expression of miR-181b in glomerular mesangial cells, establishing a link between JAK/STAT signaling and miR-181b for the first time.^24^ We have identified miR-181b-5p as a key regulator of SOCS2 in HB metastasis. To the best of our knowledge, this is the first study to provide evidence of the involvement of the miR-181b-5p/SOCS2 axis in HB metastasis. These findings are highly novel and significant since they highlight a new regulatory axis involved in HB metastasis.

EMT is a process by which epithelial cells acquire mesenchymal properties, allowing them to invade surrounding tissues and migrate to distant organs, and activation of the JAK/STAT signaling pathway can induce cellular EMT and thus promote tumor metastasis.^25^ In our study, we have shown that the overexpression of miR-181b leads to the downregulation of E-cadherin and upregulation of N-cadherin, snail, and vimentin, which are characteristic markers of EMT. Furthermore, we have demonstrated that the inhibition of miR-181b significantly suppresses EMT and tumor metastasis. Targeting miR-181b might be used to prevent HB cells from undergoing EMT and acquiring invasive properties, leading to reduced HB recurrence and metastasis.

There are several limitations of this study. Firstly, the study only focused on miR-181b and SOCS2 and did not consider other regulatory factors involved in HB metastasis. Further research could investigate the involvement of other miRNAs and regulatory pathways in HB metastasis. Secondly, the sample size is relatively small, and more extensive studies with larger sample sizes could provide a more comprehensive understanding of the role of the miR-181b/SOCS2 axis in HB metastasis.

## Conclusion

In summary, we have identified for the first time that miR-181b-5p directly targets SOCS2 in HB. In addition, we found that the biological effects of miR-181b-5p on HB metastasis were mediated through inhibition of SOCS2 and subsequent activation of the JAK2/STAT5 signaling pathway. Our research suggests that miR-181b-5p may serve as a therapeutic target for HB patients.

## Availability of data and materials

The datasets presented in this study can be found in online repositories. The names of the repository/repositories and accession number(s) are as follows: [GSE153089 AND https://www.ncbi.nlm.nih.gov/geo/query/acc.cgi?acc=GSE153089]. Other data and materials used during the current study are available from the corresponding author on reasonable request.
